# Supplementary material for: A Global Screen for Assembly State Changes of the Mitotic Proteome by SEC-SWATH-MS
Source: Cell Syst. 2020 Feb 26;10(2):133–155.e6. doi: 10.1016/j.cels.2020.01.001 (PMC7042714; doi:10.1016/j.cels.2020.01.001)

Q96Q83 | ALKB3_HUMAN | ALKBH3 ABH3 DEPC1
Monomer MW [kDa]: 33.375 Monomer expected elution fraction: 50

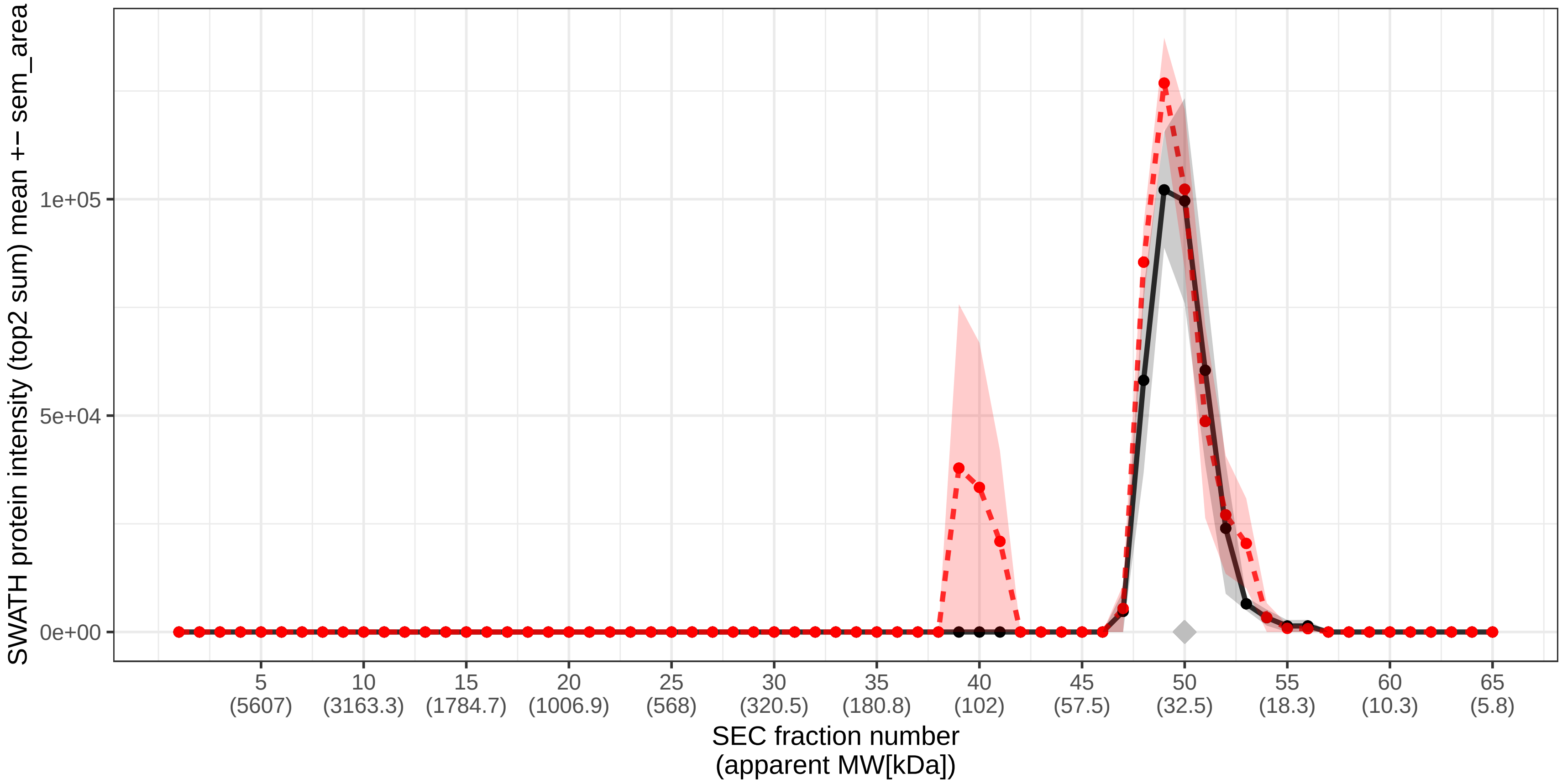

Supplement: Data S1. SEC-SWATH-MS Protein Chromatograms, Related to Figure 1 [file mmc6.zip › SECchrom_Q96Q83_ALKB3_HUMAN_ALKBH3_ABH3_DEPC1.pdf]
